# FiGS: a filter-based gene selection workbench for microarray data

**DOI:** 10.1186/1471-2105-11-50

**Published:** 2010-01-26

**Authors:** Taeho Hwang, Choong-Hyun Sun, Taegyun Yun, Gwan-Su Yi

**Affiliations:** 1 Department of Bio and Brain Engineering, KAIST, Daejeon 305-701, South Korea; 2 Department of Computer Science, KAIST, Daejeon 305-701, South Korea

## Abstract

**Background:**

The selection of genes that discriminate disease classes from microarray data is widely used for the identification of diagnostic biomarkers. Although various gene selection methods are currently available and some of them have shown excellent performance, no single method can retain the best performance for all types of microarray datasets. It is desirable to use a comparative approach to find the best gene selection result after rigorous test of different methodological strategies for a given microarray dataset.

**Results:**

FiGS is a web-based workbench that automatically compares various gene selection procedures and provides the optimal gene selection result for an input microarray dataset. FiGS builds up diverse gene selection procedures by aligning different feature selection techniques and classifiers. In addition to the highly reputed techniques, FiGS diversifies the gene selection procedures by incorporating gene clustering options in the feature selection step and different data pre-processing options in classifier training step. All candidate gene selection procedures are evaluated by the .632+ bootstrap errors and listed with their classification accuracies and selected gene sets. FiGS runs on parallelized computing nodes that capacitate heavy computations. FiGS is freely accessible at http://gexp.kaist.ac.kr/figs.

**Conclusion:**

FiGS is an web-based application that automates an extensive search for the optimized gene selection analysis for a microarray dataset in a parallel computing environment. FiGS will provide both an efficient and comprehensive means of acquiring optimal gene sets that discriminate disease states from microarray datasets.

## Background

Gene selection methods for microarray data analysis are important to identify the significant genes that distinguish disease classes and to use these selected genes as diagnostic markers in clinical decisions. Due to the significance of this matter, many gene selection methods have been introduced. Each method demonstrated a proper level of quality to predict disease states in its own test datasets, but the performance level was only partially validated in the sense that a limited number of sample datasets, gene selection algorithms, and the parameters in the test were used. It is not only difficult to choose the best performing gene selection method among many methods for a newly introduced dataset, it is also doubtful if such methods exist that can always guarantee the performance with all types of microarray datasets. A desirable approach would be to find and use a gene selection procedure that is optimized for each microarray dataset in question through a rigorous performance test of representative types of methods while varying the parameters in the methods. To complete these complex works conveniently and efficiently, the tool should automate the comprehensive tests and facilitate high-performance computing.

There have been several tools that support a comparative analysis of different gene selection methods. Prophet enables a comparison of the performance of different feature selection methods and classifiers with leave-one-out cross-validation (LOOCV) errors [1]. The procedures are automated to test the multiple classifiers but the user should select each feature selection method. Gene Expression Model Selector (GEMS) provides an automatic selection of several feature selection methods and different types of multi-category support vector machine (MC-SVM) algorithms [2]. The authors concluded that MC-SVM was the most effective classifier after a test of several different types of classifiers, including tests involving an ensemble classification method, K-nearest neighbors and neural networks. M@CBETH [3] is a tool designed to compare only classification algorithms. The aforementioned tools are useful but each has room for improvement in terms of automation, the diversity of method comparisons, or the information content in the output report.

The present study introduces FiGS, a new web-based gene selection workbench. FiGS automates the generation and evaluation of various gene selection procedures in a parallel computation environment. In addition to the well established feature selection methods and classification algorithms, FiGS uniquely incorporates several methodological options which can improve performance in certain types of datasets. Those involve the specified selection of up- or down-regulated genes in the feature selection step, feature discretization, and new feature vector formation with the addition of the expression vectors of the selected genes in classifier training step. A test using six cancer microarray datasets showed that different types of gene selection procedures should be applied to obtain the optimal gene sets for each dataset.

## Implementation

**Figure 1:**
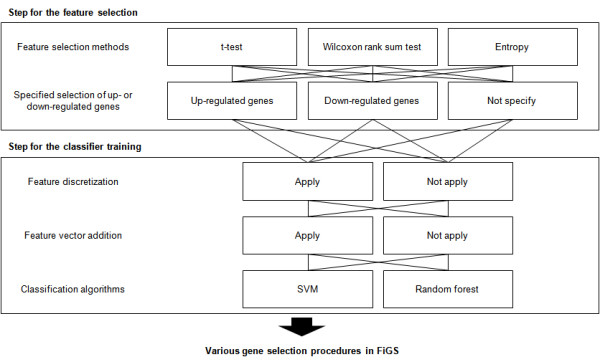
**Various gene selection procedures in FiGS**. As many as 60 different gene selection procedures can be developed by combining the feature selection methods, classification algorithms and various optional techniques. The feature vector addition technique is applied only to cases where the specified selection of up-regulated or down-regulated genes is used in the feature selection step. The range for the number of genes can be also set by users, though it is not shown here.

**Figure 2:**
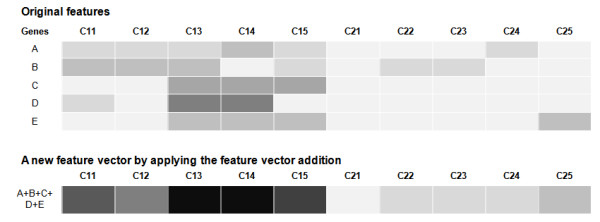
**The feature vector addition technique**. The symbol Cij is the jth sample in the ith class. The darker gray represents the higher expression values.

FiGS generates gene selection procedures having two separate steps: feature selection and classifier training (Figure 1). The feature selection step is used for the prioritization of differentially expressed genes. The distributional assumption for microarray data can change for each dataset based on the dataset size, presence of experimental artifacts, underlying gene expression setup and other factors [4]. One of our goals is to find proper statistical measures that match these unknown types of microarray data distributions automatically. To facilitate this process, three proven test statistics were selected from among a wide range of parametric and non-parametric or model-free methods. The t-test and the Wilcoxon rank sum test were included as a representative parametric and non-parametric analysis, respectively. In addition, an information gain method using an entropy-based discretization measure [5] was included as another type of non-parametric method. Using these methods, FiGS can evaluate the significance of genes with diverse input data features: the original numeric values, the rank-transformed values and discretized values. In addition, we designed a new option to specify up- or down-regulated genes for input. The genes are assigned to up- or down-regulated genes based on their differential expression in two classes. In some cases, the selection of genes in those separate groups can enhance the classification power (See Table 1 to find the examples in our test). During the classifier training step, the support vector machine (SVM) [6] and the random forest [7] methods were incorporated. These are the state-of-the-art classification algorithms that showed excellent performance in recent comparative analyses [8,9]. The features used for classifier training can be diversified in two types: the original values and the discretized values. Previous studies showed that feature discretization can considerably improve classification performance [10,11]. In addition, we developed a new form of feature vector for classifier training. It forms a new feature vector by adding the gene expression features of selected genes (Figure 2). As shown in Figure 2, it can generate an improved feature by amplifying a similar expression pattern or offsetting the outliers. The performance of classifier is evaluated by the .632+ bootstrap method [12].

**Table 1:** The best performing gene selection procedure with the .632+ bootstrap error identified by FiGS for each of the six microarray datasets.

Dataset	Feature selection method	*k*	Gene expression pattern	Feature discretization	Feature vector addition	Classifier	Error
Leukemia	Wilcoxon rank sum test	10	Down-regulated	Not apply	Not apply	SVM	0.02
Leukemia	Wilcoxon rank sum test	10	Down-regulated	Not apply	Not apply	RF	0.02
Leukemia	Wilcoxon rank sum test	10	Down-regulated	Not apply	Apply	SVM	0.02
Leukemia	Wilcoxon rank sum test	10	Down-regulated	Not apply	Apply	RF	0.02
Leukemia	Wilcoxon rank sum test	10	Down-regulated	Apply	Not apply	SVM	0.02
Leukemia	Information gain method	10	Down-regulated	Not apply	Not apply	SVM	0.02
Leukemia	Information gain method	10	Down-regulated	Not apply	Not apply	RF	0.02
Leukemia	Information gain method	10	Down-regulated	Not apply	Apply	SVM	0.02
Leukemia	Information gain method	10	Down-regulated	Not apply	Apply	RF	0.02
Leukemia	Information gain method	10	Down-regulated	Apply	Not apply	SVM	0.02
Leukemia	Information gain method	10	Down-regulated	Apply	Not apply	RF	0.02
Colon	Information gain method	30	Up-regulated	Not apply	Not apply	RF	0.11
Prostate	Information gain method	25	Total	Not apply	Not apply	RF	0.05
Adenocarcinoma	Wilcoxon rank sum test	10	Up-regulated	Not apply	Not apply	RF	0.10
Breast	Wilcoxon rank sum test	15	Down-regulated	Not apply	Apply	SVM	0.31
Breast	Information gain method	15	Down-regulated	Not apply	Apply	SVM	0.31
DLBCL	Wilcoxon rank sum test	20	Total	Not apply	Not apply	RF	0.08

*k *is the number of selected genes; and error is the .632+ bootstrap error achieved by the best performing gene selection procedure tested on 100 bootstrap samples. In the case of the leukemia and breast datasets, the multiple gene selection procedures are the best.

All algorithms in the introduced gene selection procedure are implemented in R [13]. The e1071 [14] and randomForest [15] R-package were used to implement the SVM and the random forest, respectively. The computations are parallelized using the Parallel Virtual Machine (PVM) via the rpvm [16] and snow [17] in the R-packages on a cluster of 9 nodes, each with dual quad-core Intel Xeon 2.46 GHz CPUs and 24 Gb RAM.

In the web-based user interface, FiGS takes a two-class microarray dataset as input in a tab-delimited text file. Users are allowed to run the default setup suggested by FiGS or to design their own comparative study by selecting from among the methods and techniques described above. Users can specify several desired numbers of genes to be selected. The output reports the performance of each gene selection procedure with the selected genes. The output is available through the web site or can be automatically emailed upon a user's request.

## Results

**Figure 3:**
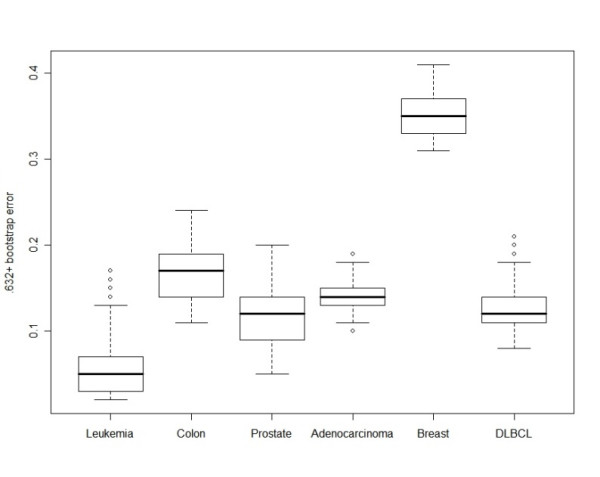
**Box plots of the .632+ bootstrap errors obtained by different gene selection procedures for each of the six cancer microarray datasets**.

Several analyses were conducted to demonstrate the necessity and usefulness of the comprehensive approach of FiGS. We tested the performance of all the possible gene selection procedures that can be generated by FiGS to six binary (two-class) microarray datasets. The microarray datasets were chosen from the literature because they have been extensively analyzed in previous publications. The six microarray datasets were as follows: leukemia (33 samples with 3051 genes) [18], colon (64 samples with 2000 genes) [19], prostate (102 samples with 6033 genes) [20], adenocarcinoma (76 samples with 9868 genes) [21], breast (77 samples with 4869 genes) [22], and diffuse B-cell lymphoma (DLBCL) (77 samples with 5469 genes) [23]. Figure 3 shows the range of .632+ bootstrap errors in the classification models generated by all the gene selection procedures for each microarray dataset. Even in the same dataset, the errors differ greatly depending on which gene selection procedure is used. The deviation between the best and worst .632+ bootstrap error for a dataset is in the range of 0.09 (adenocarcinoma) to 0.15 (leukemia). This range implies that there is a high chance of getting a much poorer classification model if an inappropriate gene selection procedure is used without caution. In addition, the best performing gene selection procedures for each datasets are different. We examined the best performing methods which produce the smallest .632+ bootstrap errors with a minimum number of genes for each dataset. The best optimized methods for each dataset are listed in Table 1 with their .632+ bootstrap errors and the number of selected genes. For the feature selection step, we found in all six cases that the model-free or non-parametric methods are preferable to the parametric t-test. According to the literature, non-parametric methods are more applicable in the analysis of microarray data, where the sample size is often small and an underlying distribution can be hardly assumed [4]. Nevertheless, some microarray datasets can be appropriate for the parametric t-test, especially studies involving large numbers of samples. Within two non-parametric methods, we can notice that there is no dominant appearance of a single method. Both Wilcoxon rank sum test and the information gain method can be used for leukemia and breast datasets. However, the best feature selection method for the adenocarcinoma and DLBCL datasets is the Wilcoxon rank sum test, and the best method for the colon and prostate datasets is the information gain method. For the classifiers, the SVM and the random forest are both competitive, though as with the feature selection methods the applicability of each method differs for each dataset. The random forest was chosen for the colon, prostate, adenocarcinoma, and DLBCL datsets; the SVM was selected for the breast dataset. There is no correlation between the types of feature selection method and classifiers among the best selected procedures. Note also that our newly developed methodological designs in FiGS are frequently selected as part of the best gene selection procedures for those datasets. Those are the strategy to select the genes in a separate group having up- or down-regulated pattern and the feature vector addition technique. All the results underscore the need for a comprehensive comparison of the many applicable procedures.

**Figure 4:**
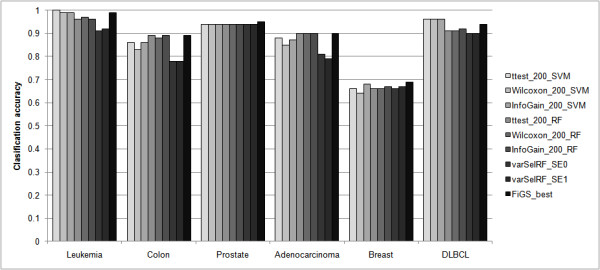
**Comparison of the best performing gene selection procedures identified by FiGS with other gene selection approaches in terms of the classification accuracy**. The names of the compared gene selection procedures are abbreviated as follows: ttest, t-test; Wilcoxon, Wilcoxon rank sum test; and InfoGain, information gain method. 200 is the number of genes to select. varSelRF_SE0 and varSelRF_SE1 are two versions of varSelRF each with the standard error (SE) term set to 0 and 1, respectively. FiGS_best is the best gene selection procedure identified by FiGS; it produces the best classification accuracy with the smallest number of genes. The classification accuracy represented in the y-axis is 1-.632+bootstrap error.

Using the same six microarray datasets, we compared the classification performance of the best gene selection procedures found in FiGS with those of typical gene selection approaches. Six gene selection procedures were fabricated by combining three feature selection methods (namely, the t-test, the Wilcoxon rank sum test, and the information gain method) with two classification algorithms (namely, the SVM and random forest). The number of genes to select was set to 200 on the assumption that 200 is a sufficiently large number for those six methods to give their best performance. While substantially optimizing the number of genes, FiGS could find better, or at least comparable, level of classification accuracy than that achieved by the six methods (Figure 4) with much fewer genes. The number of genes that satisfies the level of accuracy for FiGS as shown in Figure 4 is 50 for leukemia, 30 for colon, 25 for prostate, 10 for adenocarcinoma, 15 for breast, and 70 for DLBCL. We also tried to compare the performance of FiGS with recently published methods. However, it is hard to fairly and comprehensively evaluate the performance of different gene selection methods due to the different setups of the experimental design, datasets, and the performance definitions [2]. Of the various methods, we found varSelRF, a gene selection method based on random forest backward feature elimination [15], as an appropriate method that can be readily compared with FiGS. The classification performances of varSelRF for all datasets that FiGS has tested except the DLBCL dataset are available in [8]. For the DLBCL dataset, we obtained the results by using the varSelRF tools provided by the R-package. Figure 4 shows the results of two versions of varSelRF. The best optimized gene selection procedures by FiGS outperform the two different modes of varSelRF in terms of the classification accuracy for all tested datasets. Although varSelRF automatically selected very few genes on the basis of its internal optimization strategy, the classification performance seems to have been compromised.

During our comprehensive comparison of the various gene selection procedures in FiGS for the six real microarray datasets, we found no single method is dominant in its performance. Thus, a comprehensive search for various gene selection procedures is both necessary and useful. Although the gene selection methods included in FiGS are not a complete spectrum of all available methods, the set of methods selected in FiGS seems reasonable. Most methods in FiGS were selected almost equally frequently in the final best gene selection procedure for the different datasets. The best optimized gene selection procedures selected by FiGS for the tested cancer microarray datasets outperformed other typical approaches and recently developed methods.

## Conclusion

Finding and using a proper gene selection procedure specific to a given microarray dataset is necessary and useful. FiGS facilitates this procedure. It is an easy-to-use workbench that selects promising genes for disease classification on the basis of a rigorous and comprehensive test of various gene selection procedures for a given microarray dataset. FiGS helps researchers efficiently and conveniently determine biomarker candidates for clinical application with a greater level of accuracy and reliability.

## Availability and requirements

**Project name: **FiGS

**Project homepage: **http://gexp.kaist.ac.kr/figs

**Operating system(s): **Platform independent (web-based application).

**Programming language: **R, Perl and Java script.

**Other requirements: **A web brower.

**License: **None for usage.

**Any restrictions to use by non-academics: **None.

## Authors' contributions

GSY conceived and designed this study and interpreted the results. TH designed and carried out the analyses, and wrote the R-based source codes and the web-based interfaces. TH and GSY participated in writing the manuscript. CHS and TY contributed to building and testing the infrastructures for parallel computing and the benchmark test.
